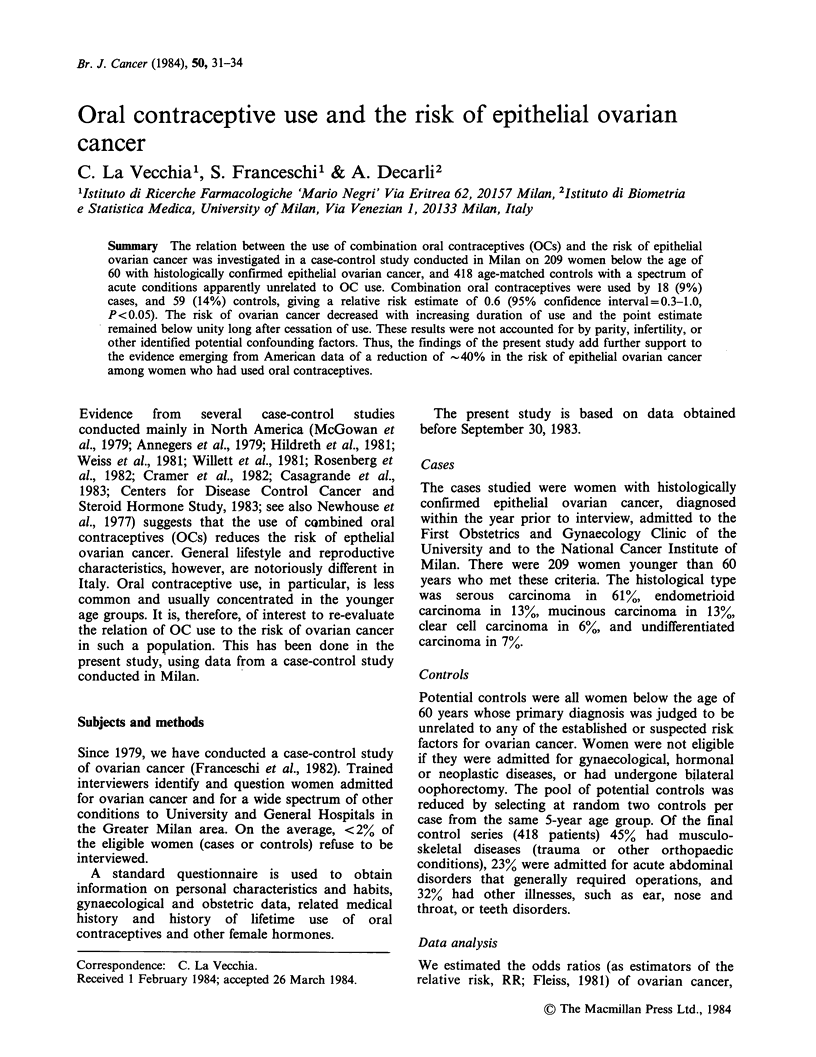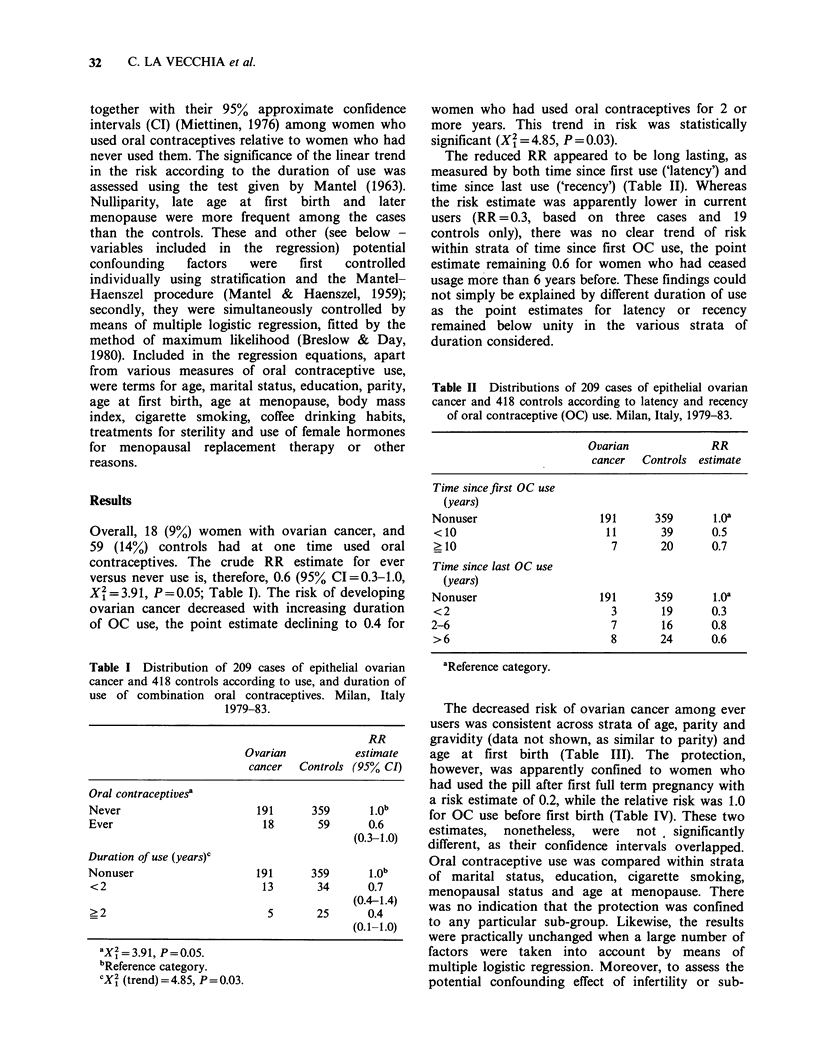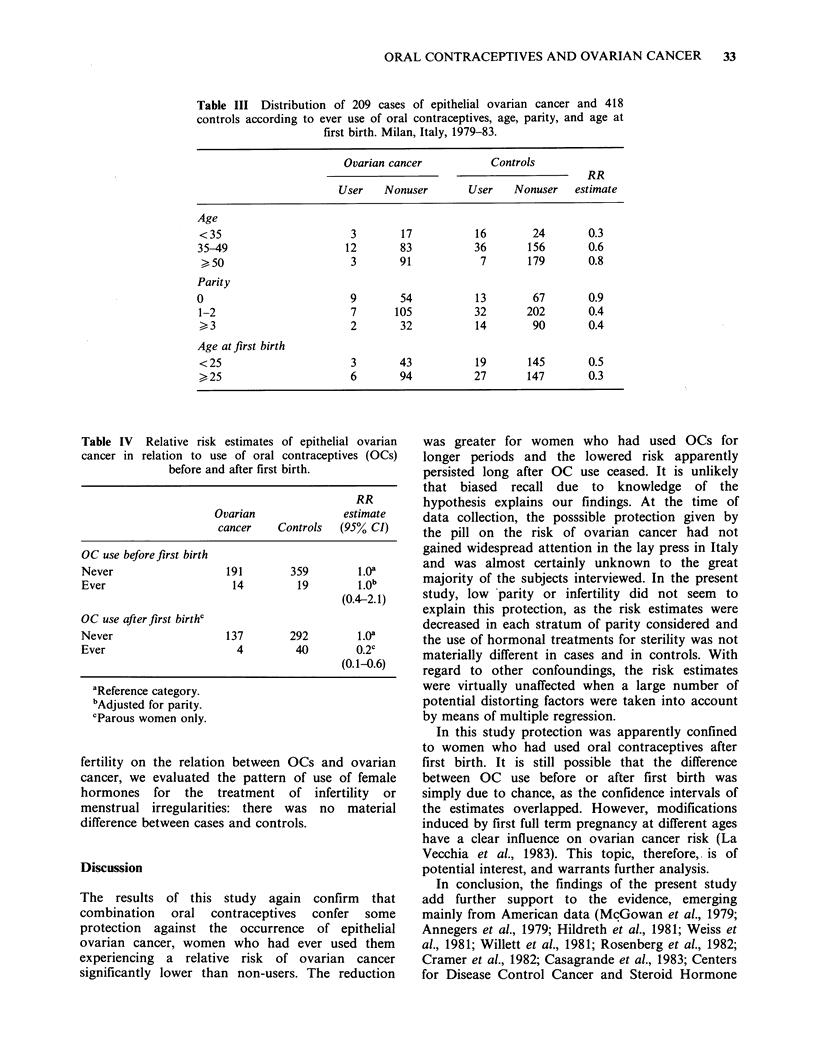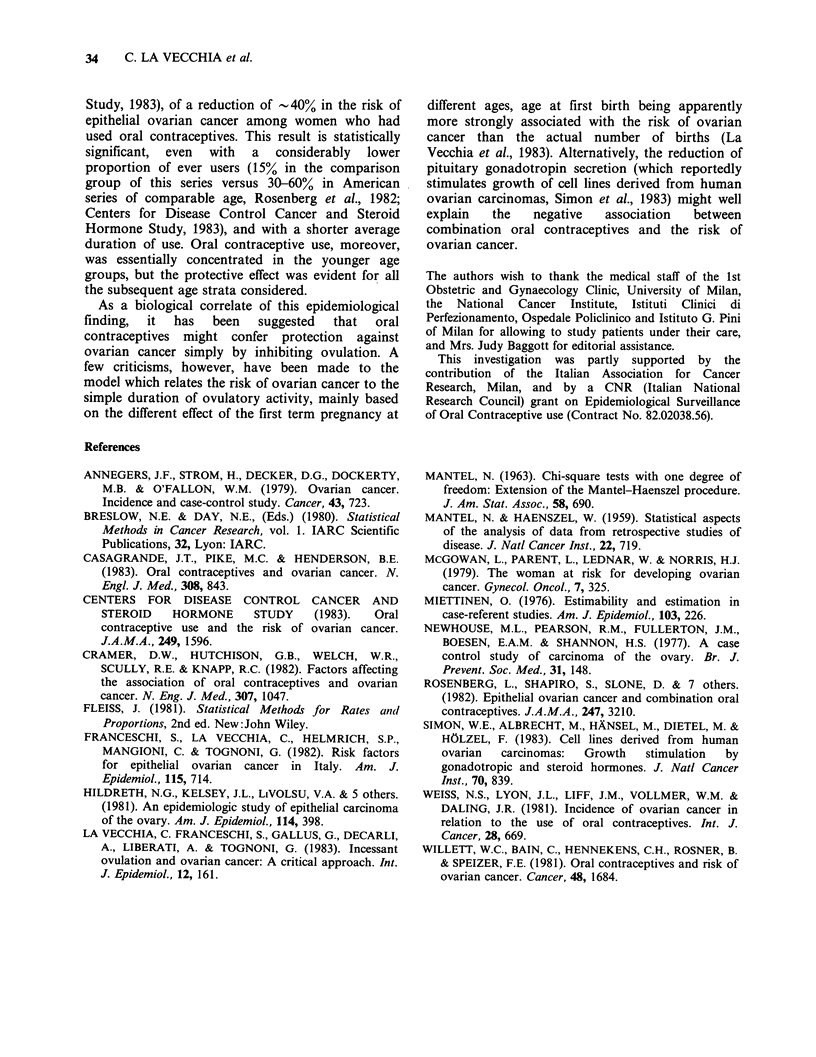# Oral contraceptive use and the risk of epithelial ovarian cancer.

**DOI:** 10.1038/bjc.1984.136

**Published:** 1984-07

**Authors:** C. La Vecchia, S. Franceschi, A. Decarli

## Abstract

The relation between the use of combination oral contraceptives (OCs) and the risk of epithelial ovarian cancer was investigated in a case-control study conducted in Milan on 209 women below the age of 60 with histologically confirmed epithelial ovarian cancer, and 418 age-matched controls with a spectrum of acute conditions apparently unrelated to OC use. Combination oral contraceptives were used by 18 (9%) cases, and 59 (14%) controls, giving a relative risk estimate of 0.6 (95% confidence interval = 0.3-1.0, P less than 0.05). The risk of ovarian cancer decreased with increasing duration of use and the point estimate remained below unity long after cessation of use. These results were not accounted for by parity, infertility, or other identified potential confounding factors. Thus, the findings of the present study add further support to the evidence emerging from American data of a reduction of approximately 40% in the risk of epithelial ovarian cancer among women who had used oral contraceptives.


*Br. J. Cancer* (1984), **50**, 31–34

### Oral contraceptive use and the risk of epithelial ovarian cancer

C. La Vecchia[1], S. Franceschi[1] & A. Decarli[2]

[1]*Istituto di Ricerche Farmacologiche 'Mario Negri' Via Eritrea 62, 20157 Milan,* [2]*Istituto di Biometria e Statistica Medica, University of Milan, Via Venezian 1, 20133 Milan, Italy*

**Summary** The relation between the use of combination oral contraceptives (OCs) and the risk of epithelial ovarian cancer was investigated in a case-control study conducted in Milan on 209 women below the age of 60 with histologically confirmed epithelial ovarian cancer, and 418 age-matched controls with a spectrum of acute conditions apparently unrelated to OC use. Combination oral contraceptives were used by 18 (9%) cases, and 59 (14%) controls, giving a relative risk estimate of 0.6 (95% confidence interval = 0.3–1.0, $P < 0.05$). The risk of ovarian cancer decreased with increasing duration of use and the point estimate remained below unity long after cessation of use. These results were not accounted for by parity, infertility, or other identified potential confounding factors. Thus, the findings of the present study add further support to the evidence emerging from American data of a reduction of ~40% in the risk of epithelial ovarian cancer among women who had used oral contraceptives.

Evidence from several case-control studies conducted mainly in North America (McGowan *et al.*, 1979; Annegers *et al.*, 1979; Hildreth *et al.*, 1981; Weiss *et al.*, 1981; Willett *et al.*, 1981; Rosenberg *et al.*, 1982; Cramer *et al.*, 1982; Casagrande *et al.*, 1983; Centers for Disease Control Cancer and Steroid Hormone Study, 1983; see also Newhouse *et al.*, 1977) suggests that the use of combined oral contraceptives (OCs) reduces the risk of epthelial ovarian cancer. General lifestyle and reproductive characteristics, however, are notoriously different in Italy. Oral contraceptive use, in particular, is less common and usually concentrated in the younger age groups. It is, therefore, of interest to re-evaluate the relation of OC use to the risk of ovarian cancer in such a population. This has been done in the present study, using data from a case-control study conducted in Milan.

### Subjects and methods

Since 1979, we have conducted a case-control study of ovarian cancer (Franceschi *et al.*, 1982). Trained interviewers identify and question women admitted for ovarian cancer and for a wide spectrum of other conditions to University and General Hospitals in the Greater Milan area. On the average, <2% of the eligible women (cases or controls) refuse to be interviewed.

A standard questionnaire is used to obtain information on personal characteristics and habits, gynaecological and obstetric data, related medical history and history of lifetime use of oral contraceptives and other female hormones.

The present study is based on data obtained before September 30, 1983.

### *Cases*

The cases studied were women with histologically confirmed epithelial ovarian cancer, diagnosed within the year prior to interview, admitted to the First Obstetrics and Gynaecology Clinic of the University and to the National Cancer Institute of Milan. There were 209 women younger than 60 years who met these criteria. The histological type was serous carcinoma in 61%, endometrioid carcinoma in 13%, mucinous carcinoma in 13%, clear cell carcinoma in 6%, and undifferentiated carcinoma in 7%.

### *Controls*

Potential controls were all women below the age of 60 years whose primary diagnosis was judged to be unrelated to any of the established or suspected risk factors for ovarian cancer. Women were not eligible if they were admitted for gynaecological, hormonal or neoplastic diseases, or had undergone bilateral oophorectomy. The pool of potential controls was reduced by selecting at random two controls per case from the same 5-year age group. Of the final control series (418 patients) 45% had musculo-skeletal diseases (trauma or other orthopaedic conditions), 23% were admitted for acute abdominal disorders that generally required operations, and 32% had other illnesses, such as ear, nose and throat, or teeth disorders.

### *Data analysis*

We estimated the odds ratios (as estimators of the relative risk, RR; Fleiss, 1981) of ovarian cancer,

Correspondence: C. La Vecchia.
Received 1 February 1984; accepted 26 March 1984.

© The Macmillan Press Ltd., 1984

together with their 95% approximate confidence intervals (CI) (Miettinen, 1976) among women who used oral contraceptives relative to women who had never used them. The significance of the linear trend in the risk according to the duration of use was assessed using the test given by Mantel (1963). Nulliparity, late age at first birth and later menopause were more frequent among the cases than the controls. These and other (see below – variables included in the regression) potential confounding factors were first controlled individually using stratification and the Mantel–Haenszel procedure (Mantel & Haenszel, 1959); secondly, they were simultaneously controlled by means of multiple logistic regression, fitted by the method of maximum likelihood (Breslow & Day, 1980). Included in the regression equations, apart from various measures of oral contraceptive use, were terms for age, marital status, education, parity, age at first birth, age at menopause, body mass index, cigarette smoking, coffee drinking habits, treatments for sterility and use of female hormones for menopausal replacement therapy or other reasons.

### Results

Overall, 18 (9%) women with ovarian cancer, and 59 (14%) controls had at one time used oral contraceptives. The crude RR estimate for ever versus never use is, therefore, 0.6 (95% CI=0.3–1.0, $X_1^2=3.91$, $P=0.05$; Table I). The risk of developing ovarian cancer decreased with increasing duration of OC use, the point estimate declining to 0.4 for women who had used oral contraceptives for 2 or more years. This trend in risk was statistically significant ($X_1^2=4.85$, $P=0.03$).

**Table I** Distribution of 209 cases of epithelial ovarian cancer and 418 controls according to use, and duration of use of combination oral contraceptives. Milan, Italy 1979–83.

| | *Ovarian cancer* | *Controls* | *RR estimate (95% CI)* |
|---|---|---|---|
| *Oral contraceptives*[a] | | | |
| Never | 191 | 359 | 1.0[b] |
| Ever | 18 | 59 | 0.6 (0.3–1.0) |
| *Duration of use (years)*[c] | | | |
| Nonuser | 191 | 359 | 1.0[b] |
| <2 | 13 | 34 | 0.7 (0.4–1.4) |
| ≧2 | 5 | 25 | 0.4 (0.1–1.0) |

[a]$X_1^2=3.91$, $P=0.05$.

[b]Reference category.

[c]$X_1^2$ (trend)$=4.85$, $P=0.03$.

The reduced RR appeared to be long lasting, as measured by both time since first use ('latency') and time since last use ('recency') (Table II). Whereas the risk estimate was apparently lower in current users (RR=0.3, based on three cases and 19 controls only), there was no clear trend of risk within strata of time since first OC use, the point estimate remaining 0.6 for women who had ceased usage more than 6 years before. These findings could not simply be explained by different duration of use as the point estimates for latency or recency remained below unity in the various strata of duration considered.

**Table II** Distributions of 209 cases of epithelial ovarian cancer and 418 controls according to latency and recency of oral contraceptive (OC) use. Milan, Italy, 1979–83.

| | *Ovarian cancer* | *Controls* | *RR estimate* |
|---|---|---|---|
| *Time since first OC use (years)* | | | |
| Nonuser | 191 | 359 | 1.0[a] |
| <10 | 11 | 39 | 0.5 |
| ≧10 | 7 | 20 | 0.7 |
| *Time since last OC use (years)* | | | |
| Nonuser | 191 | 359 | 1.0[a] |
| <2 | 3 | 19 | 0.3 |
| 2–6 | 7 | 16 | 0.8 |
| >6 | 8 | 24 | 0.6 |

[a]Reference category.

The decreased risk of ovarian cancer among ever users was consistent across strata of age, parity and gravidity (data not shown, as similar to parity) and age at first birth (Table III). The protection, however, was apparently confined to women who had used the pill after first full term pregnancy with a risk estimate of 0.2, while the relative risk was 1.0 for OC use before first birth (Table IV). These two estimates, nonetheless, were not significantly different, as their confidence intervals overlapped. Oral contraceptive use was compared within strata of marital status, education, cigarette smoking, menopausal status and age at menopause. There was no indication that the protection was confined to any particular sub-group. Likewise, the results were practically unchanged when a large number of factors were taken into account by means of multiple logistic regression. Moreover, to assess the potential confounding effect of infertility or sub-

**Table III** Distribution of 209 cases of epithelial ovarian cancer and 418 controls according to ever use of oral contraceptives, age, parity, and age at first birth. Milan, Italy, 1979–83.

| | *Ovarian cancer* | | *Controls* | | *RR* |
|---|---|---|---|---|---|
| | *User* | *Nonuser* | *User* | *Nonuser* | *estimate* |
| *Age* | | | | | |
| <35 | 3 | 17 | 16 | 24 | 0.3 |
| 35–49 | 12 | 83 | 36 | 156 | 0.6 |
| ⩾50 | 3 | 91 | 7 | 179 | 0.8 |
| *Parity* | | | | | |
| 0 | 9 | 54 | 13 | 67 | 0.9 |
| 1–2 | 7 | 105 | 32 | 202 | 0.4 |
| ⩾3 | 2 | 32 | 14 | 90 | 0.4 |
| *Age at first birth* | | | | | |
| <25 | 3 | 43 | 19 | 145 | 0.5 |
| ⩾25 | 6 | 94 | 27 | 147 | 0.3 |

**Table IV** Relative risk estimates of epithelial ovarian cancer in relation to use of oral contraceptives (OCs) before and after first birth.

| | *Ovarian cancer* | *Controls* | *RR estimate (95% CI)* |
|---|---|---|---|
| *OC use before first birth* | | | |
| Never | 191 | 359 | 1.0[a] |
| Ever | 14 | 19 | 1.0[b] (0.4–2.1) |
| *OC use after first birth*[c] | | | |
| Never | 137 | 292 | 1.0[a] |
| Ever | 4 | 40 | 0.2[c] (0.1–0.6) |

[a]Reference category.
[b]Adjusted for parity.
[c]Parous women only.

fertility on the relation between OCs and ovarian cancer, we evaluated the pattern of use of female hormones for the treatment of infertility or menstrual irregularities: there was no material difference between cases and controls.

### Discussion

The results of this study again confirm that combination oral contraceptives confer some protection against the occurrence of epithelial ovarian cancer, women who had ever used them experiencing a relative risk of ovarian cancer significantly lower than non-users. The reduction was greater for women who had used OCs for longer periods and the lowered risk apparently persisted long after OC use ceased. It is unlikely that biased recall due to knowledge of the hypothesis explains our findings. At the time of data collection, the posssible protection given by the pill on the risk of ovarian cancer had not gained widespread attention in the lay press in Italy and was almost certainly unknown to the great majority of the subjects interviewed. In the present study, low parity or infertility did not seem to explain this protection, as the risk estimates were decreased in each stratum of parity considered and the use of hormonal treatments for sterility was not materially different in cases and in controls. With regard to other confoundings, the risk estimates were virtually unaffected when a large number of potential distorting factors were taken into account by means of multiple regression.

In this study protection was apparently confined to women who had used oral contraceptives after first birth. It is still possible that the difference between OC use before or after first birth was simply due to chance, as the confidence intervals of the estimates overlapped. However, modifications induced by first full term pregnancy at different ages have a clear influence on ovarian cancer risk (La Vecchia *et al.*, 1983). This topic, therefore, is of potential interest, and warrants further analysis.

In conclusion, the findings of the present study add further support to the evidence, emerging mainly from American data (McGowan *et al.*, 1979; Annegers *et al.*, 1979; Hildreth *et al.*, 1981; Weiss *et al.*, 1981; Willett *et al.*, 1981; Rosenberg *et al.*, 1982; Cramer *et al.*, 1982; Casagrande *et al.*, 1983; Centers for Disease Control Cancer and Steroid Hormone

Study, 1983), of a reduction of ~40% in the risk of epithelial ovarian cancer among women who had used oral contraceptives. This result is statistically significant, even with a considerably lower proportion of ever users (15% in the comparison group of this series versus 30–60% in American series of comparable age, Rosenberg *et al.*, 1982; Centers for Disease Control Cancer and Steroid Hormone Study, 1983), and with a shorter average duration of use. Oral contraceptive use, moreover, was essentially concentrated in the younger age groups, but the protective effect was evident for all the subsequent age strata considered.

As a biological correlate of this epidemiological finding, it has been suggested that oral contraceptives might confer protection against ovarian cancer simply by inhibiting ovulation. A few criticisms, however, have been made to the model which relates the risk of ovarian cancer to the simple duration of ovulatory activity, mainly based on the different effect of the first term pregnancy at different ages, age at first birth being apparently more strongly associated with the risk of ovarian cancer than the actual number of births (La Vecchia *et al.*, 1983). Alternatively, the reduction of pituitary gonadotropin secretion (which reportedly stimulates growth of cell lines derived from human ovarian carcinomas, Simon *et al.*, 1983) might well explain the negative association between combination oral contraceptives and the risk of ovarian cancer.

The authors wish to thank the medical staff of the 1st Obstetric and Gynaecology Clinic, University of Milan, the National Cancer Institute, Istituti Clinici di Perfezionamento, Ospedale Policlinico and Istituto G. Pini of Milan for allowing to study patients under their care, and Mrs. Judy Baggott for editorial assistance.

This investigation was partly supported by the contribution of the Italian Association for Cancer Research, Milan, and by a CNR (Italian National Research Council) grant on Epidemiological Surveillance of Oral Contraceptive use (Contract No. 82.02038.56).

### References

ANNEGERS, J.F., STROM, H., DECKER, D.G., DOCKERTY, M.B. & O'FALLON, W.M. (1979). Ovarian cancer. Incidence and case-control study. *Cancer*, **43**, 723.

BRESLOW, N.E. & DAY, N.E., (Eds.) (1980). *Statistical Methods in Cancer Research*, vol. 1. IARC Scientific Publications, **32**, Lyon: IARC.

CASAGRANDE, J.T., PIKE, M.C. & HENDERSON, B.E. (1983). Oral contraceptives and ovarian cancer. *N. Engl. J. Med.*, **308**, 843.

CENTERS FOR DISEASE CONTROL CANCER AND STEROID HORMONE STUDY (1983). Oral contraceptive use and the risk of ovarian cancer. *J.A.M.A.*, **249**, 1596.

CRAMER, D.W., HUTCHISON, G.B., WELCH, W.R., SCULLY, R.E. & KNAPP, R.C. (1982). Factors affecting the association of oral contraceptives and ovarian cancer. *N. Eng. J. Med.*, **307**, 1047.

FLEISS, J. (1981). *Statistical Methods for Rates and Proportions*, 2nd ed. New: John Wiley.

FRANCESCHI, S., LA VECCHIA, C., HELMRICH, S.P., MANGIONI, C. & TOGNONI, G. (1982). Risk factors for epithelial ovarian cancer in Italy. *Am. J. Epidemiol.*, **115**, 714.

HILDRETH, N.G., KELSEY, J.L., LIVOLSU, V.A. & 5 others. (1981). An epidemiologic study of epithelial carcinoma of the ovary. *Am. J. Epidemiol.*, **114**, 398.

LA VECCHIA, C. FRANCESCHI, S., GALLUS, G., DECARLI, A., LIBERATI, A. & TOGNONI, G. (1983). Incessant ovulation and ovarian cancer: A critical approach. *Int. J. Epidemiol.*, **12**, 161.

MANTEL, N. (1963). Chi-square tests with one degree of freedom: Extension of the Mantel–Haenszel procedure. *J. Am. Stat. Assoc.*, **58**, 690.

MANTEL, N. & HAENSZEL, W. (1959). Statistical aspects of the analysis of data from retrospective studies of disease. *J. Natl Cancer Inst.*, **22**, 719.

McGOWAN, L., PARENT, L., LEDNAR, W. & NORRIS, H.J. (1979). The woman at risk for developing ovarian cancer. *Gynecol. Oncol.*, **7**, 325.

MIETTINEN, O. (1976). Estimability and estimation in case-referent studies. *Am. J. Epidemiol.*, **103**, 226.

NEWHOUSE, M.L., PEARSON, R.M., FULLERTON, J.M., BOESEN, E.A.M. & SHANNON, H.S. (1977). A case control study of carcinoma of the ovary. *Br. J. Prevent. Soc. Med.*, **31**, 148.

ROSENBERG, L., SHAPIRO, S., SLONE, D. & 7 others. (1982). Epithelial ovarian cancer and combination oral contraceptives. *J.A.M.A.*, **247**, 3210.

SIMON, W.E., ALBRECHT, M., HÄNSEL, M., DIETEL, M. & HÖLZEL, F. (1983). Cell lines derived from human ovarian carcinomas: Growth stimulation by gonadotropic and steroid hormones. *J. Natl Cancer Inst.*, **70**, 839.

WEISS, N.S., LYON, J.L., LIFF, J.M., VOLLMER, W.M. & DALING, J.R. (1981). Incidence of ovarian cancer in relation to the use of oral contraceptives. *Int. J. Cancer*, **28**, 669.

WILLETT, W.C., BAIN, C., HENNEKENS, C.H., ROSNER, B. & SPEIZER, F.E. (1981). Oral contraceptives and risk of ovarian cancer. *Cancer*, **48**, 1684.